# Association between ambient temperature and increased total length of hospital stay of patients with cardiopulmonary disease in Hong Kong

**DOI:** 10.3389/fpubh.2024.1411137

**Published:** 2024-12-18

**Authors:** Chenxiang Long, Shengyu Guo, Ping Tian, Yingying Sun

**Affiliations:** ^1^College of Biology and Engineering, Guizhou Medical University, Guiyang, China; ^2^Department of Economics and Management, Changsha University, Changsha, China; ^3^School of Public Health, Guizhou Medical University, Guiyang, China; ^4^Medical Affairs Department, Emergency General Hospital, Beijing, China

**Keywords:** length of hospital, cold, heat, cardiopulmonary disease, temperature extremes

## Abstract

**Background:**

While temperature extremes have been shown to be associated with an increased risk of hospital admissions, evidence of their impact on the length of hospital stay, which may capture the lingering effects of temperature extremes, is scarce.

**Objectives:**

We aimed to evaluate the association between daily variation in ambient temperature and daily variation in daily total length of stay (daily TLOS), a composite measure encompassing the daily count of hospital admissions and their corresponding length of hospital stay among cardiopulmonary patients. Additionally, we quantified the burden of TLOS attributable to non-optimal temperatures among Hong Kong’s older adult population.

**Methods:**

We used a generalized linear regression with a distributed lag non-linear model to estimate the association between ambient temperature and daily TLOS. The analysis used 13 years of time-series data (1998–2010) on daily temperature and hospital admissions for cardiopulmonary diseases through accident and emergency departments among Hong Kong’s older adult population. We quantified the attributable risk of TLOS by calculating the temperature-related days of hospital stay and the attributable fraction (AF).

**Results:**

We recorded a total of 4,095,722 hospital stay days for cardiovascular patients and 4,492,697 days for respiratory patients. We found that both cold and heat were associated with increased TLOS for cardiopulmonary disease. The temperature-related AF was 11.5% (95% empirical CI: 5.3–17.2%) for cardiovascular disease, corresponding to an annual increase of 36,174 days (95% empirical CI: 15,286–57,018). For respiratory disease, the AF was 10.7% (95% empirical CI: 7.1–13.9%), equating to an annual increase of 36,897 days (95% empirical CI: 24,949–49,024) days annually.

**Conclusion:**

Extreme temperatures were associated with increased TLOS for cardiopulmonary patients in Hong Kong’s older adult population. These findings highlight the need for hospitals to prepare in advance for extreme temperature events by implementing specific measures in terms of human resources and medical resources. In addition, the results provide valuable scientific evidence to support public health policies and inform hospital planning and management.

## 1 Introduction

Cardiopulmonary disease is the leading cause of death and years of life lost globally (1). In 2016, an estimated 17.6 million people worldwide died from cardiovascular disease, and approximately 3.5 million deaths were attributed to chronic respiratory diseases. These numbers are expected to steadily increase (1).

In addition to mortality, cardiopulmonary disease poses a significant burden on healthcare systems due to rising hospitalization rates. The prevalence of cardiopulmonary disease has been increasing dramatically, driven by advancements in biomedical research and improvements in life expectancy (2). At the same time, higher medical care costs place a greater financial burden on patients, and prolonged exposure to healthcare environments may result in a potential decline in the health status of patients. From a healthcare system perspective, longer stays can lead to higher bed occupancy rates, reduced availability for new admissions, and increased demand for medical staff and resources.

A large body of evidence has convincingly established the presence of an association between non-optimal temperature and cardiopulmonary mortality or morbidity (3–8). While most previous studies have shown the short-term (e.g., daily) impact of temperature extremes on the numbers of cardiopulmonary mortality or morbidity, mostly focusing on dichotomous events (e.g., hospitalized or not) but ignoring the subsequent impacts. The impact of temperature may linger over time, resulting in a longer hospital stay for the patients. Given the ongoing reality of climate change (9) and the expected rise in the proportion of people suffering from cardiopulmonary disease, understanding the health burden of non-optimal temperatures on cardiopulmonary disease is of significant public health importance.

The length of hospital stay provides a complementary indicator for evaluating the health burden of a patient with a non-optimal temperature. No existing research has incorporated the length of hospital stay into examining the short-term association between temperature and hospital admissions. We aimed to estimate the association between daily ambient temperature and a composite measure of daily count of hospital admissions and their corresponding length of hospital stay, namely daily total length of stay (daily TLOS), of cardiopulmonary disease among individuals aged 65 or over in Hong Kong. We also quantified the total burden of daily TLOS attributable to non-optimal temperature for cardiovascular and respiratory diseases, respectively.

## 2 Materials and methods

### 2.1 Outcome measure

We obtained the number of hospital admissions from the Hospital Authority Corporate Data Warehouse. The data warehouse is run by the Hospital Authority, records hospital admissions from all publicly funded hospitals that provide 24-h emergency services, and covers approximately 90% of hospital beds in Hong Kong (10). These data comprised date of birth, sex, date of admission, date of discharge, admission source, and principal diagnosis International Classification of Diseases (ICD) code. We restricted our analysis to admissions through accident and emergency departments with principal diagnoses of cardiovascular disease (ICD-9: 390–459) and respiratory disease (ICD-9: 460–519) from 1998 to 2010. The length of hospital stay for each admission was the number of days between the date of discharge and the date of admission. The daily TLOS was the product of the total number of emergency hospital admissions on a particular day and their corresponding lengths of stay, stratified by sex (male and female) of the patients. Informed consent from patients was not required as we only used aggregated data.

### 2.2 Meteorological and air pollution data

Daily mean ambient temperature and relative humidity data were obtained from the Hong Kong Observatory. We defined extreme and moderate cold as days with mean temperature in the ≤1st and 10th percentiles of daily mean temperatures over the study period, respectively, whereas extreme and moderate heat was defined as days with temperatures in the ≥99th and 90th percentiles of daily mean temperatures, respectively (7).

We also obtained daily 24-h average concentrations of particulate matter with aerodynamic diameter ≤10 μm (PM_10_) and nitrogen dioxide (NO_2_) from 10 general monitoring stations maintained by the Hong Kong Environmental Protection Department (Supplementary Figure S1). To calculate the daily mean concentration of air pollution, we averaged the daily concentrations of air pollution across the 10 monitoring stations (11).

### 2.3 Statistical analysis

Given that the daily TLOS of cardiovascular and respiratory diseases followed a normal distribution (Supplementary Figure S2), we estimated the association between daily ambient temperature and daily TLOS using a generalized linear regression (12, 13). We used a distributed-lag non-linear model by creating a cross-basis term to describe the non-linear and delayed effects of temperature (14). Specifically, the cross-basis term of temperature included a natural cubic spline for temperature with 3 degrees of freedom and a natural cubic spline with three internal knots placed equally on the log scale of the lag day. A maximum lag day of 21 was used to fully capture the delayed effects of temperature (15). We used the lowest quasi-Akaike score (Q-AIC) to guide the selection of degrees of freedom for temperature and lag day. In order to control for long-term and seasonal trends, a natural cubic B-spline function with 7 degrees of freedom per year was included in the model. We also controlled for a day of the week, public holiday, relative humidity with a natural cubic spline with 3 degrees of freedom, and air pollutants of PM_10_ and NO_2_ simultaneously at a 2-day moving average (lag0-1) in the model (16). We identified the optimal temperature as the temperature with the minimum daily TLOS. Cold and heat effects were computed with reference to the optimal temperature (17). Our sensitivity analysis compared our effect estimates of the daily TLOS with those from a standard quasi-Poisson regression using the daily count of hospital admissions (18).

We further quantified TLOS attributable to non-optimal temperature by summing the contributions from all the days in the series using the optimal temperature as the reference. Then, we calculated the attributable fraction by dividing the TLOS by the total attributable length of stay (18). The empirical confidence intervals (empirical CIs) for an attributable length of hospital stay and attributable fraction were obtained by Monte Carlo simulations simulating 5,000 samples from a multivariate normal distribution of the coefficients (18).

All analyses were conducted in R, version 3.5.1. We used the ‘dlnm’ package to fit the distributed-lag non-linear model to estimate the association between daily temperature and 149 daily TLOS. We calculated TLOS attributable to non-optimal temperature by modifying the ‘attrdl’ function (18).

## 3 Results

### 3.1 Description statistics

**Figure 1:**
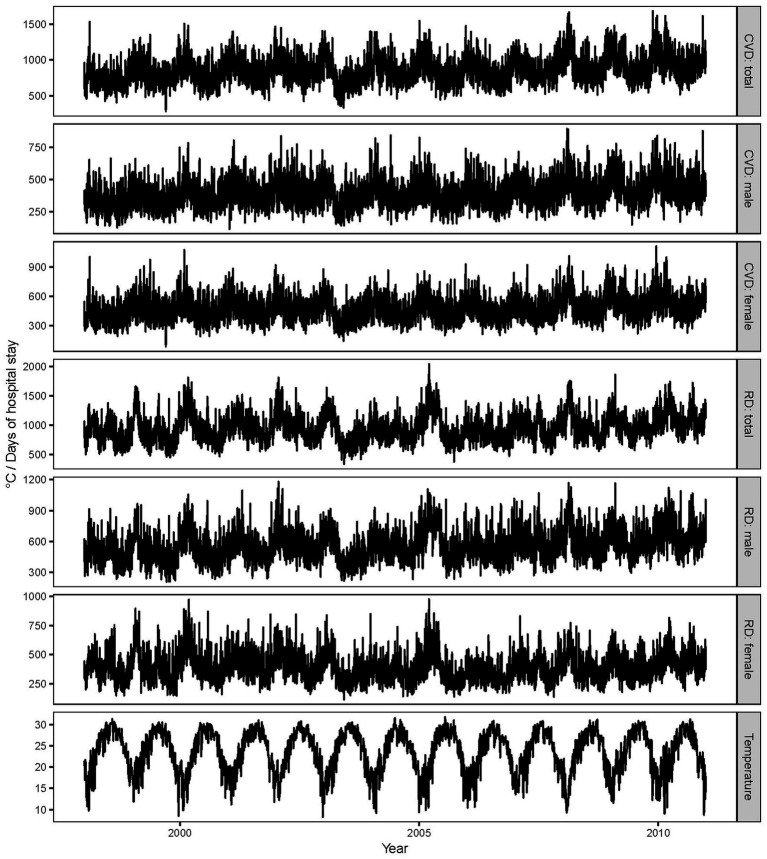
Time-series plot for ambient temperature and total length of hospital stay. CVD, cardiovascular; RD, respiratory disease.

During the study period, over 4 million days of hospital stay were recorded for both cardiovascular and respiratory diseases. On average, the daily TLOS was 863 and 946 for cardiovascular and respiratory patients, respectively, corresponding to 120 and 146 hospital admissions (Table 1). Female individuals were more likely to be admitted to the hospital and contributed to larger daily TLOS for cardiovascular disease, while male individuals tended to be hospitalized for respiratory disease. The daily 24-h mean concentration of air pollution was 52.1 μg/m^3^ for PM_10_ and 57.2 μg/m^3^ for NO_2_. The average daily mean temperature was 23.5°C, and the mean relative humidity was 78.0% (Table 1). Daily length of hospital stay showed seasonal trends, which were higher in the cold season (October to March) than in the warm season (April to September) (Figure 1).

**Table 1:** Summary statistics of count and length of hospital stay for cardiopulmonary patients via emergency departments, air pollution, and meteorological conditions in Hong Kong, 1998–2010.

Variable	Mean (SD)	Percentile				
		Min	P_25_	P_50_	P_75_	Max
Length of hospital stay (day)						
Cardiovascular	863 (192)	286	725	844	982	1,680
Male	391 (112)	113	312	380	459	897
Female	471 (127)	82	380	459	547	1,112
Respiratory	946 (230)	333	783	919	1,084	2,040
Male	556 (153)	208	447	539	648	1,181
Female	390 (120)	114	302	372	458	975
Number of hospital admissions (n)						
Cardiovascular	120 (25)	45	103	116	134	251
Male	55 (13)	22	46	53	62	119
Female	65 (14)	20	55	63	74	143
Respiratory	146 (34)	55	121	141	165	300
Male	86 (21)	26	70	83	98	200
Female	60 (16)	23	49	58	69	153
Air pollutants (μg/m^3^)						
PM_10_	52.1 (28.3)	12.0	29.9	46.5	68.7	572.2
NO_2_	57.2 (20.4)	9.8	42.5	54.7	68.4	167.0
Meteorological conditions						
Ambient temperature	23.5 (5.0)	8.2	19.6	24.7	27.8	31.8
Relative humidity (%)	78.0 (10.3)	27.5	73.4	79.1	84.9	98.1

SD, standard deviation; Min, minimum; P_25_, 25th percentile; P_50_, 50th percentile; P_75_, 75th percentile; Max, maximum; PM_10_, particulate matter with aerodynamic diameter < 10 μm; NO_2_, nitrogen dioxide.

### 3.2 Modeling results

**Figure 2:**
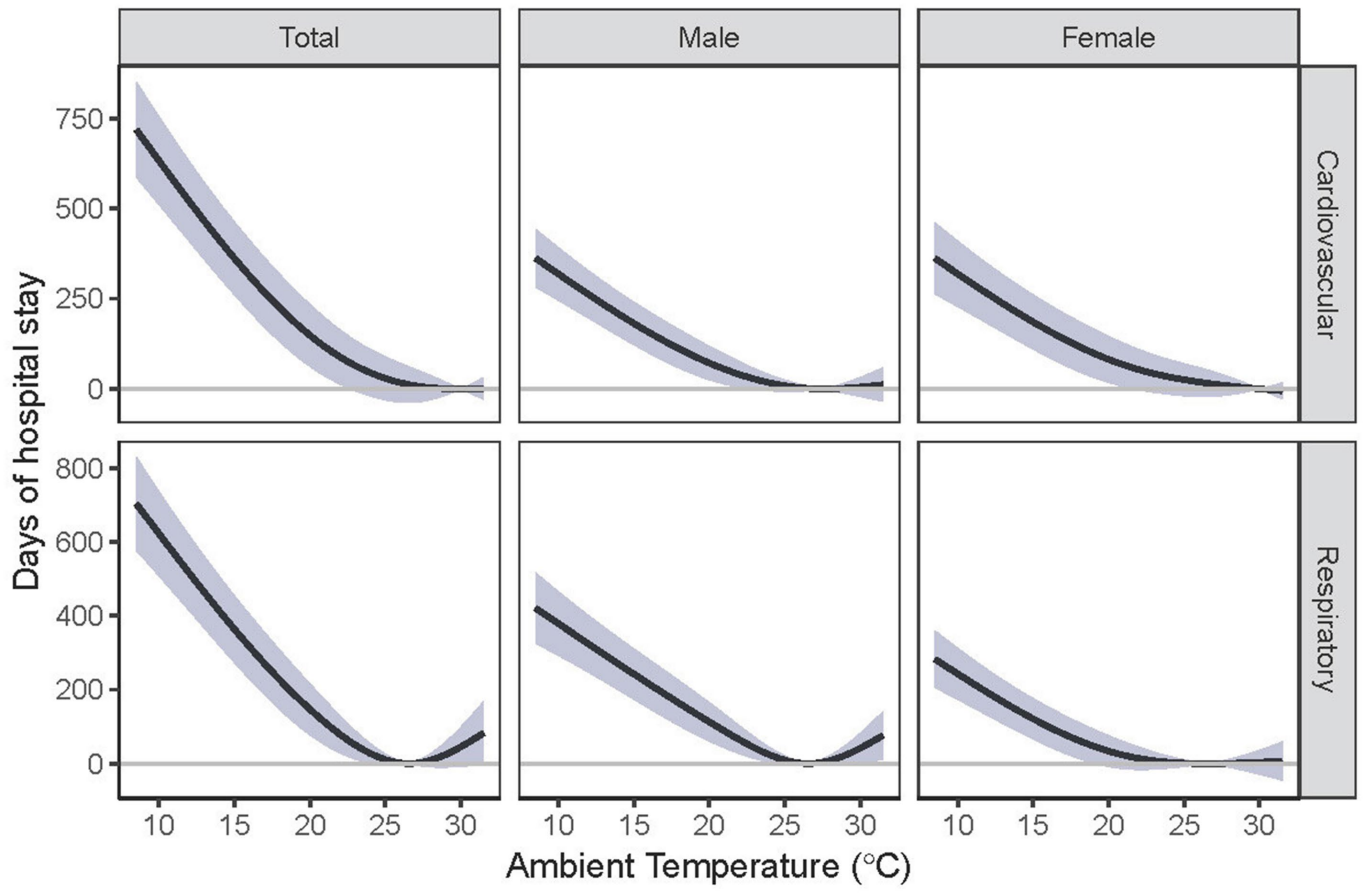
Cumulative relationship between ambient temperature and total length of hospital stay among Hong Kong’s older adult population. Estimates were compared with the corresponding optimal temperature.

**Figure 3:**
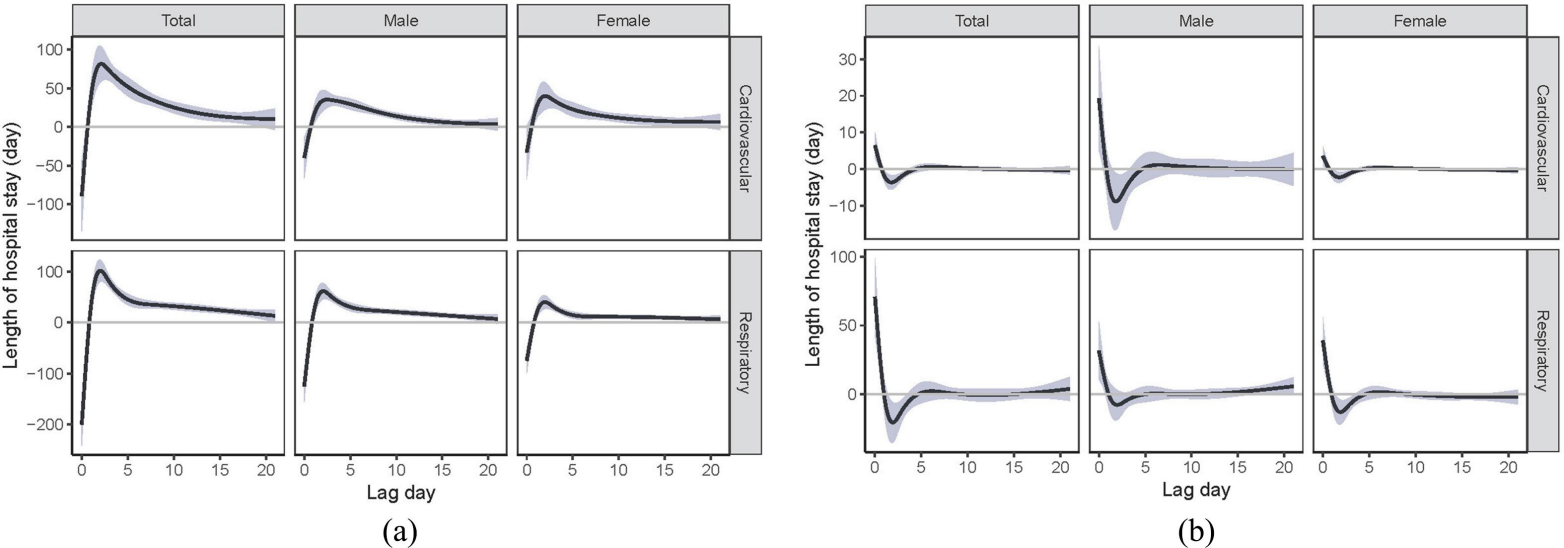
Lag-specific effects of extreme cold **(A)** and heat **(B)** on total length of hospital stay for cardiopulmonary disease over 21 days of lag among Hong Kong’s older adult population. Extreme cold was defined as the 1st percentile of temperature (11.6°C); extreme heat was defined as the 99th percentile of temperature (30.4°C). Estimates were compared with the corresponding optimal temperature.

We consistently observed a reverse J-shaped relation between daily temperature and daily TLOS for cardiovascular and respiratory diseases and by sex, with increased days of hospital stay associated with both heat and cold (Figure 2). We found that cold effects on daily TLOS were delayed for 2–3 days and lasted for 2–3 weeks, but heat effects were immediate and lasted for less than 5 days, and these lag patterns for cold and heat effects were similar for cardiovascular and respiratory diseases (Figure 3). Similar lag patterns were also found for cold and heat across sex.

Table 2 shows the annual length of hospital stay among patients with cardiopulmonary disease associated with cold and heat over multiple lag days. Regarding cardiovascular disease, compared with the optimal temperature, extreme cold (1st, 11.6°C) was associated with an increase of 42 (95% CI: 33–50) days of hospital stay over 21 days annually, and moderate cold (10th, 16.4°C) was associated with an increase of 23 (95% CI: 15–30) days of hospital stay. Extreme and moderate cold effects on daily TLOS for respiratory disease were comparable with cardiovascular disease. For example, extreme cold (1st, 11.6°C) was associated with an increase of 41 (95% CI: 33–49) days of hospital stay annually compared with the optimal temperature. The cold effects for male and female individuals were comparable among cardiovascular patients but showed heterogeneity among patients with respiratory disease, with stronger estimates for male participants compared to female participants. For example, extreme cold (1st, 11.6°C) was associated with an increase of 26 (95% CI: 20–32) days of hospital stay annually for male participants, while female participants experienced a rise of 16 (95% CI: 11–20) days of hospital stay.

**Table 2:** Annual length of hospital stay (days) associated with cold and heat over multiple lag days among Hong Kong’s older adult population with cardiopulmonary disease, 1998–2010.

Diseases	Group	Optimal temperature	Lag day	Extreme cold^1^	Moderate cold^2^	Moderate heat^3^	Extreme heat^4^
Cardiovascular	Total	30.0	0-1	−4 (−7, 0)	−4 (−7, −1)	−1 (−1, 0)	0 (0, 1)
		30.0	0–7	23 (18, 28)	12 (8, 16)	0 (−1, 0)	0 (0, 0)
		30.0	0–14	35 (29, 42)	19 (13, 25)	0 (−1, 0)	0 (0, 1)
		30.0	0–21	42 (33, 50)	23 (15, 30)	0 (−1, 1)	0 (−1, 1)
	Male	27.5	0–1	−2 (−4, 0)	−2 (−4, 0)	1 (0, 1)	1 (0, 2)
		27.5	0–7	12 (9, 15)	6 (3, 8)	0 (−1, 1)	0 (−1, 2)
		27.5	0–14	18 (15, 22)	10 (6, 13)	0 (−1, 1)	1 (−1, 2)
		27.5	0–21	21 (16, 26)	11 (7, 16)	0 (−1, 2)	1 (−2, 3)
	Female	30.0	0–1	−1 (−3, 2)	−1 (−3, 1)	0 (−1, 0)	0 (0, 0)
		30.0	0–7	12 (8, 15)	6 (3, 9)	0 (0, 0)	0 (0, 0)
		30.0	0–14	17 (12, 22)	10 (5, 14)	0 (0, 0)	0 (0, 0)
		30.0	0–21	21 (15, 28)	12 (6, 17)	0 (0, 1)	0 (−1, 0)
Respiratory	Total	26.5	0–1	−13 (−16, −10)	−9 (−12, −7)	4 (2, 5)	5 (3, 8)
		26.5	0–7	15 (10, 19)	8 (4, 11)	2 (0, 4)	3 (0, 6)
		26.5	0–14	31 (25, 37)	17 (12, 22)	2 (−1, 4)	3 (0, 7)
		26.5	0–21	41 (33, 49)	23 (16, 30)	2 (−1, 6)	4 (−1, 9)
	Male	26.5	0–1	−8 (−11, −6)	−6 (−8, −4)	2 (1, 3)	3 (1, 4)
		26.5	0–7	9 (6, 13)	5 (2, 8)	1 (0, 2)	2 (0, 4)
		26.5	0–14	20 (15, 25)	11 (7, 15)	1 (−1, 3)	2 (−1, 5)
		26.5	0–21	26 (20, 32)	16 (11, 21)	2 (0, 5)	4 (0, 7)
	Female	26.5	0–1	−5 (−7, −3)	−3 (−5, −2)	2 (1, 3)	3 (1, 4)
		26.5	0–7	5 (3, 8)	3 (1, 5)	1 (0, 2)	2 (0, 3)
		26.5	0–14	11 (7, 15)	6 (3, 9)	1 (−1, 2)	1 (−1, 3)
		26.5	0–21	16 (11, 20)	7 (3, 11)	0 (−2, 2)	0 (−3, 3)

1 The 1st percentile of temperature (11.6°C) compared to the optimal temperature.

2 The 10th percentile of temperature (16.4°C) compared to the optimal temperature.

3 The 90th percentile of temperature (29.4°C) compared to the optimal temperature.

4 The 99th percentile of temperature (30.4°C) compared to the optimal temperature.

Heat was associated with increased TLOS for respiratory disease only, with risk estimates notably smaller compared to those for cold. For example, extreme heat (99th, 30.4°C) was associated with a 5-day increase in hospital stay (95% CI: 3–8) at the moving average of the temperature on the admission date and 1 day before (lag0-1) for respiratory disease annually.

To compare with the count of hospital stays, we calculated the relative risk of cold and heat on emergency hospital admissions, as shown in Supplementary Table S1. For example, compared to the optimal temperature, the cumulative relative risk of respiratory disease hospitalizations over 21 days was associated with 1.75 (95% CI: 1.63–1.88) for extreme cold and 1.33 (95% CI: 1.25–1.42) for moderate cold. Moderate heat and extreme heat were associated with respiratory disease hospitalizations with relative risks of 1.05 (95% CI: 1.03–1.07) and 1.04 (95% CI: 1.02–1.05), respectively. We also observed a reverse J-shaped exposure-response curve between temperature and the count of hospital admissions, with both cold and heat associated with an increased risk of hospitalizations for cardiopulmonary diseases (Supplementary Figure S3).

We further quantified TLOS attributable to non-optimal temperature, as indicated in Table 3. Overall, 11.5% (95% empirical CI: 5.3–17.2%) of TLOS for cardiovascular disease was attributed to non-optimal temperature, corresponding to 36,174 (95% empirical CI: 15,286–57,018) days of hospital stay annually in Hong Kong’s older adult population. Non-optimal temperature was responsible for 10.7% (95% empirical CI: 7.1–13.9%) of TLOS for respiratory disease, which equals 36,897 (95% empirical CI: 24,949–49,024) days of hospital stay annually. Cold was responsible for the majority of the burden of TLOS for both cardiovascular and respiratory diseases. For example, the attributable fraction of cold was 9.9% (95% empirical CI: 6.2–13.3%) for respiratory disease, while heat only accounted for 0.8% (95% empirical CI: −0.3 to 1.9%). We also found that the attributable risk for TLOS for respiratory disease was higher in male participants than female participants (attributable fraction: 13.2% versus 7.1%).

**Table 3:** Attributable number and attributable fraction associated with non-optimal temperature for length of hospital stay among Hong Kong’s older adult population.

Diseases	Optimal temperature	Attributable number annually			Attributable fraction		
		Total	Cold^1^	Heat^2^	Tota^l^	Cold	Heat
Cardiovascular	30.0	36,174 (15,286, 57,018)	36,172 (16,295, 53,851)	2 (−96, 89)	11.5 (5.3, 17.2)	11.5 (5.2, 17.7)	0.0 (0.0, 0.0)
Male	27.5	17,502 (9,343, 25,733)	17,249 (9,007, 26,236)	252 (−1,357, 1,655)	12.2 (6.6, 17.7)	12.1 (6.6, 18.3)	0.2 (−0.9, 1.3)
Female	30.0	20,586 (6,350, 34,950)	20,604 (6,637, 35,782)	−18 (−90, 54)	12.0 (3.2, 20.4)	12.0 (3.3, 19.9)	0.0 (0.0, 0.0)
Respiratory	26.5	36,897 (24,949, 49,024)	34,177 (22,547, 45,859)	2,720 (−1,003, 6,505)	10.7 (7.1, 13.9)	9.9 (6.2, 13.3)	0.8 (−0.3, 1.9)
Male	26.5	26,759 (19,158, 35,247)	24,266 (15,049, 33,522)	2,493 (−83, 5,222)	13.2 (8.8, 17.4)	11.9 (7.7, 16.2)	1.2 (−0.1, 2.6)
Female	26.5	10,138 (2,968, 17,234)	9,911 (2,281, 17,255)	227 (−2,059, 2,526)	7.1 (2.3, 12.0)	7.0 (2.0, 12.3)	0.2 (−1.5, 1.7)

1 Cold represented days with a temperature lower than the optimal temperature.

2 Heat represented days with a temperature higher than the optimal temperature.

## 4 Discussion

To the best of our knowledge, this is the first study to investigate the association between temperature and daily TLOS, and we found that both cold and heat were associated with increased days of TLOS. The non-optimal temperature was responsible for over 10% of TLOS among cardiopulmonary patients in Hong Kong’s older adult population, and the majority of the burden was due to cold. Our findings add to existing literature that shows that days of non-optimal temperature were associated with increased days of TLOS.

Previous studies mainly focused on the count of hospital admissions to assess the impact of non-optimal temperatures. They did not account for the length of hospital stay for each admission (3, 19, 20). Our daily TLOS indicator, a composite measure of daily counts of hospital admissions and their corresponding hospital stay duration, is considered a more informative and differentiated measurement for assessing the impact of non-optimal temperatures and hospital service utilization than counts of hospital admissions alone. Given that climate change is occurring (9) and emergency department overcrowding in public hospitals is continuing, knowing the extent of the increase in days of hospital stay associated with non-optimal temperature is critical to better understand the impact of temperature and patient flows.

Our study also assessed the association between temperature and counts of hospital admission, and we found that extreme temperatures were associated with a higher risk of hospital admission for cardiovascular and respiratory diseases. Our findings were consistent with previous studies, which reported that temperature extremes were associated with an increased risk of hospitalizations (3, 20–22). For example, a study in 213 US counties with a total of 12.5 million Medicare beneficiaries found that each 10°F increase in daily temperature was associated with a 4.3% increase in respiratory emergency hospitalizations (3). A study in Ontario, Canada, with a total of 1.4 million coronary heart disease hospitalizations, reported that extreme heat (the 99th percentile) was associated with an increase in coronary heart disease hospitalizations by 6% (21) (Table 4).

**Table 4:** The comparison with other epidemiological studies in other areas is listed in the table.

Location	Time period	Diseases studied	Main findings
United States (3)	1999–2008	Respiratory diseases in the Medicare population	Each 10°F increase in daily temperature is associated with a 4.3% increase in same-day emergency hospitalizations for respiratory diseases.
Ontario, Canada (21)	1996–2013	Coronary heart disease	There is a 9% increase in daily hospitalizations for CHD on cold days (1st percentile of temperature) and a 6% increase on hot days (99th percentile).
Hong Kong, China	1998–2010	Cardiopulmonary diseases in older adult population	Both cold and heat are associated with increased total length of hospital stay (TLOS). 11.5% of TLOS for cardiovascular disease and 10.7% for respiratory disease attributable to non-optimal temperature.

This study has important implications. Emergency department crowding is a major global public health problem that impairs the quality of care and patient safety and satisfaction (23–26). The inability to move admitted patients from the emergency department to an inpatient bed is one of the most frequent reasons for emergency department crowding (27, 28). Balancing inpatient admissions and discharges is reported to be one of the effective ways to minimize emergency department crowding (29). Our study revealed that non-optimal temperatures increased inpatient bed occupancy, which accounted for over 10% of TLOS annually. These findings may help understand patient flows and provide guidance for hospital management. These findings may also enrich our understanding of the impact of temperature.

Our analysis reveals distinct gender-specific lag patterns in the temporal responses of cardiovascular and respiratory diseases to cold and heat exposure. These distinct lag patterns for cold and heat exposure were consistent across both sexes and attributed to biological, behavioral, and exposure-related factors. Biological differences between males and females may also contribute to their varying susceptibility to temperature-related health outcomes. For instance, hormonal differences may affect the thermoregulatory system, with females generally having a higher metabolic rate, which could influence their response to temperature changes. Additionally, lung function and respiratory system structure can vary by sex, with males typically having larger airways and greater lung capacity, which might provide some protection against respiratory diseases exacerbated by temperature extremes. Behavioral differences also play a role in how individuals are exposed to temperature variation. Men are more likely to engage in outdoor occupations and activities, which could increase their exposure to extreme temperatures. Conversely, women may spend more time indoors, which could offer some protection (30).

The findings of this study have significant implications for medical policy and decision-making, particularly in the context of climate change and its impact on public health. Policymakers might consider these factors when planning healthcare resource allocation and emergency preparedness (31). Firstly, hospitals need to have more flexible resource management ability. Hospitals need to allocate additional staff and beds to manage the influx of patients during periods of extreme temperatures, including cross-training staff to handle increased patient loads or establishing partnerships with other healthcare facilities to ensure adequate care capacity. Secondly, hospitals can develop emergency protocols for extreme temperature events, similar to those used for natural disasters. For example, an early warning system can alert hospital staff of impending temperature extremes, formulate patient discharge planning in advance, and increase the availability of healthcare resources. Thirdly, hospitals need to invest in healthcare infrastructure that can withstand and adapt to the changing climate. This could involve the installation of more efficient heating, ventilation, and air conditioning systems in hospitals (32).

This study also has some limitations. First, exposure measurement errors for ambient temperature might exist. We used one representative fixed-site ambient temperature monitoring station located in urban areas to represent the whole population rather than individual direct measurements. However, we expect the exposure misclassification would be non-differential and, on average, tend to bias our results toward the null hypothesis of no association. Second, there might be a decreasing trend for the length of hospital stay due to medical advancement and healthcare professionals’ motivations to shorten it to cope with the increasing demand for inpatient beds. However, we adopted a time-series study that compared daily variation in ambient temperature and daily variation in total length of hospital stay so that the long-term trend of the length of hospital stay would not introduce bias for the risk estimates of temperature on the length of hospital stay. Finally, our data were somewhat outdated, and we could not obtain the most recent data, limiting the generalizability of our findings to more recent contexts.

## 5 Conclusion

In conclusion, non-optimal temperatures were associated with increased hospital stay durations among cardiopulmonary patients in Hong Kong’s older adult population. These findings provide valuable scientific evidence for hospital planning, management, and public health policies, particularly in the context of climate change and an aging population.

## Data Availability

Publicly available datasets were analyzed in this study. This data can be found here: https://www3.ha.org.hk/data.
