# Supplementary material for: Association between ambient temperature and increased total length of hospital stay of patients with cardiopulmonary disease in Hong Kong
Source: Front Public Health. 2024 Dec 18;12:1411137. doi: 10.3389/fpubh.2024.1411137 (PMC11688489; doi:10.3389/fpubh.2024.1411137)
Supplement: Supplementary file 1 [file Data_Sheet_1.docx]

Supplementary material

**Ambient Temperature Associated with Increased Total Length of Hospital Stay of Patients with Cardiopulmonary Disease in Hong Kong**

**Table of contents**

**Table S1.** Relative risk of cold and heat on emergency hospital admissions for cardiovascular and respiratory diseases over multiple lag days in Hong Kong, 1998-2010.

**Fig. S1.** Distribution of general air monitoring stations (n=10) and the Hong Kong Observatory.

**Fig. S2.** Distribution of length of emergency hospital stay for cardiovascular and respiratory diseases.

**Fig. S3.** Cumulative relationship between ambient temperature and count of hospital admissions for cardiovascular and respiratory diseases among Hong Kong’s elderly population.

**Table S1. Relative risk of cold and heat on emergency hospital admissions for cardiovascular and respiratory diseases over multiple lag days in Hong Kong, 1998-2010.**

| Diseases | Group | Optimum temperature | Lag day | Extreme cold | Moderate cold | Moderate heat | Extreme heat |
| --- | --- | --- | --- | --- | --- | --- | --- |
| Cardiovascular | Total | 30.0 | 0-1 | 0.94 (0.91, 0.97) | 0.94 (0.91, 0.97) | 1.00 (1.00, 1.00) | 1.00 (1.00, 1.00) |
|  |  | 30.0 | 0-7 | 1.34 (1.28, 1.40) | 1.18 (1.13, 1.23) | 1.00 (0.99, 1.00) | 1.00 (1.00, 1.01) |
|  |  | 30.0 | 0-14 | 1.66 (1.57, 1.77) | 1.33 (1.26, 1.40) | 1.00 (0.99, 1.00) | 1.00 (1.00, 1.01) |
|  |  | 30.0 | 0-21 | 1.89 (1.75, 2.04) | 1.43 (1.34, 1.54) | 1.00 (0.99, 1.00) | 1.00 (1.00, 1.01) |
|  | Male | 30.0 | 0-1 | 0.90 (0.86, 0.95) | 0.91 (0.87, 0.95) | 1.00 (1.00, 1.01) | 1.00 (0.99, 1.00) |
|  |  | 30.0 | 0-7 | 1.38 (1.29, 1.46) | 1.18 (1.12, 1.25) | 1.00 (1.00, 1.01) | 1.00 (0.99, 1.01) |
|  |  | 30.0 | 0-14 | 1.74 (1.60, 1.90) | 1.36 (1.26, 1.46) | 1.00 (1.00, 1.01) | 1.00 (0.99, 1.01) |
|  |  | 30.0 | 0-21 | 2.03 (1.82, 2.26) | 1.49 (1.35, 1.65) | 1.00 (0.99, 1.01) | 1.00 (0.99, 1.01) |
|  | Female | 30.0 | 0-1 | 0.97 (0.93, 1.01) | 0.97 (0.93, 1.00) | 1.00 (0.99, 1.00) | 1.00 (1.00, 1.01) |
|  |  | 30.0 | 0-7 | 1.31 (1.24, 1.39) | 1.17 (1.11, 1.24) | 1.00 (0.99, 1.00) | 1.01 (1.00, 1.01) |
|  |  | 30.0 | 0-14 | 1.60 (1.48, 1.73) | 1.31 (1.22, 1.40) | 1.00 (0.99, 1.00) | 1.01 (1.00, 1.01) |
|  |  | 30.0 | 0-21 | 1.78 (1.61, 1.98) | 1.38 (1.26, 1.52) | 1.00 (0.99, 1.00) | 1.01 (1.00, 1.02) |
| Respiratory | Total | 27.0 | 0-1 | 0.80 (0.77, 0.82) | 0.83 (0.81, 0.85) | 1.05 (1.03, 1.07) | 1.04 (1.02, 1.05) |
|  |  | 27.0 | 0-7 | 1.15 (1.11, 1.20) | 1.07 (1.04, 1.11) | 1.04 (1.01, 1.06) | 1.02 (1.01, 1.04) |
|  |  | 27.0 | 0-14 | 1.48 (1.40, 1.56) | 1.22 (1.17, 1.28) | 1.02 (0.99, 1.05) | 1.01 (0.99, 1.03) |
|  |  | 27.0 | 0-21 | 1.75 (1.63, 1.88) | 1.33 (1.25, 1.42) | 1.03 (0.98, 1.07) | 1.01 (0.99, 1.04) |
|  | Male | 27.0 | 0-1 | 0.80 (0.78, 0.83) | 0.83 (0.81, 0.86) | 1.04 (1.02, 1.07) | 1.03 (1.02, 1.05) |
|  |  | 27.0 | 0-7 | 1.17 (1.11, 1.22) | 1.08 (1.04, 1.13) | 1.04 (1.01, 1.07) | 1.03 (1.01, 1.04) |
|  |  | 27.0 | 0-14 | 1.49 (1.40, 1.59) | 1.25 (1.18, 1.32) | 1.02 (0.99, 1.06) | 1.01 (0.99, 1.04) |
|  |  | 27.0 | 0-21 | 1.74 (1.60, 1.89) | 1.37 (1.27, 1.47) | 1.05 (1.00, 1.10) | 1.03 (0.99, 1.06) |
|  | Female | 30.0 | 0-1 | 0.75 (0.71, 0.78) | 0.79 (0.76, 0.82) | 1.01 (1.00, 1.01) | 0.99 (0.98, 1.00) |
|  |  | 30.0 | 0-7 | 1.11 (1.04, 1.18) | 1.04 (0.98, 1.10) | 1.00 (1.00, 1.01) | 0.99 (0.99, 1.00) |
|  |  | 30.0 | 0-14 | 1.43 (1.32, 1.56) | 1.17 (1.08, 1.27) | 1.00 (1.00, 1.01) | 1.00 (0.99, 1.00) |
|  |  | 30.0 | 0-21 | 1.78 (1.59, 1.99) | 1.29 (1.17, 1.43) | 1.00 (0.99, 1.01) | 1.00 (0.99, 1.01) |

The 1st percentile of temperature (11.6^o^C) compared to the optimum temperature.

The 10th percentile of temperature (16.4^o^C) compared to the optimum temperature.

The 90th percentile of temperature (29.4 ^o^C) compared to the optimum temperature.

The 99th percentile of temperature (30.4 ^o^C) compared to the optimum temperature.


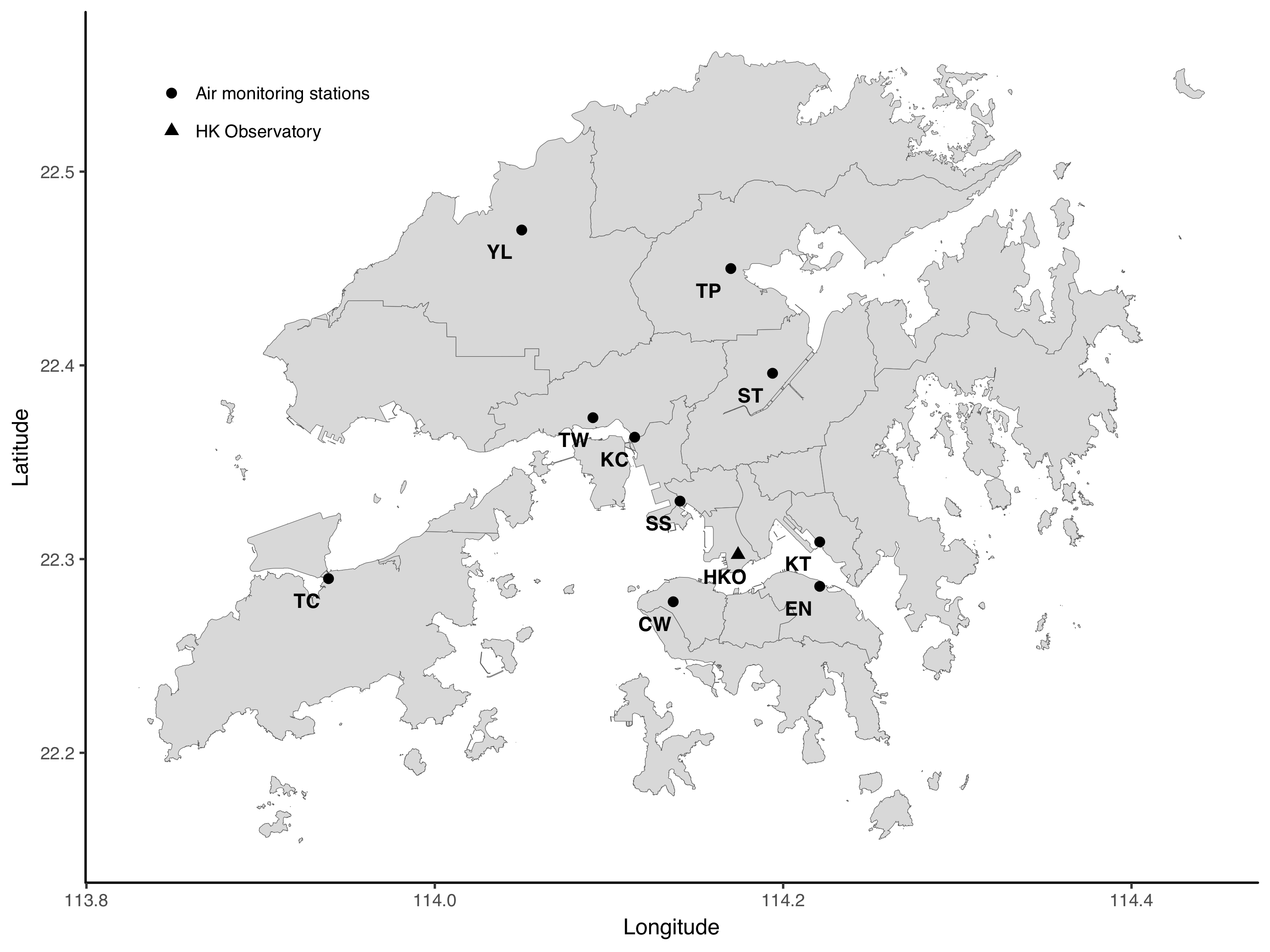


**Fig. S1. Distribution of general air monitoring stations (n=10) and the Hong Kong Observatory.**

**Fig. S2. Distribution of length of emergency hospital stay for cardiovascular and respiratory diseases.**

**Fig. S3. Cumulative relationship between ambient temperature and count of hospital admissions for cardiovascular and respiratory diseases among Hong Kong’s elderly population.**
